# DIST: direct imputation of summary statistics for unmeasured SNPs

**DOI:** 10.1093/bioinformatics/btt500

**Published:** 2013-08-28

**Authors:** Donghyung Lee, T. Bernard Bigdeli, Brien P. Riley, Ayman H. Fanous, Silviu-Alin Bacanu

**Affiliations:** Department of Psychiatry, Virginia Institute for Psychiatric and Behavioral Genetics, Virginia Commonwealth University, Richmond, Virginia 23298, USA

## Abstract

**Motivation:** Genotype imputation methods are used to enhance the resolution of genome-wide association studies, and thus increase the detection rate for genetic signals. Although most studies report all univariate summary statistics, many of them limit the access to subject-level genotypes. Because such an access is required by all genotype imputation methods, it is helpful to develop methods that impute summary statistics without going through the interim step of imputing genotypes. Even when subject-level genotypes are available, due to the substantial computational cost of the typical genotype imputation, there is a need for faster imputation methods.

**Results:** Direct Imputation of summary STatistics (DIST) imputes the summary statistics of untyped variants without first imputing their subject-level genotypes. This is achieved by (i) using the conditional expectation formula for multivariate normal variates and (ii) using the correlation structure from a relevant reference population. When compared with genotype imputation methods, DIST (i) requires only a fraction of their computational resources, (ii) has comparable imputation accuracy for independent subjects and (iii) is readily applicable to the imputation of association statistics coming from large pedigree data. Thus, the proposed application is useful for a fast imputation of summary results for (i) studies of unrelated subjects, which (a) do not provide subject-level genotypes or (b) have a large size and (ii) family association studies.

**Availability and implementation:** Pre-compiled executables built under commonly used operating systems are publicly available at http://code.google.com/p/dist/.

**Contact:** dlee4@vcu.edu

**Supplementary information:** Supplementary data are available at *Bioinformatics* online.

## 1 INTRODUCTION

Genome-wide association studies (GWASs) have been successful in detecting associations between genetic variants and complex diseases (Hindorff *et al.*, 2009). However, GWASs genotype only a fraction of the tens of millions of single nucleotide polymorphisms (SNPs) found in the human genome. To increase resolution, and thus the detection rate for genetic signals, researchers proposed imputing genotypes at numerous untyped (unmeasured) SNPs (de Bakker *et al.*, 2006; Li *et al.*, 2009; Marchini and Howie, 2010).

Most commonly used genotype imputation tools, e.g. IMPUTE2 (Howie *et al.*, 2009), MACH (Li *et al.*, 2010), BIMBAM (Servin and Stephens, 2007) and BEAGLE (Browning and Browning, 2007), are based on Hidden Markov Models (HMMs). Although these methods are accurate, due to their need for haplotypic phasing of all subjects in the study, they are extremely burdensome computationally. Their computational burden would become even more extreme with the ever increasing size of studies and reference panels. Other genotype imputation methods [PLINK (Purcell *et al.*, 2007), SNPMSTAT (Lin *et al.*, 2008), UNPHASED (Dudbridge, 2008) and TUNA (Wen and Nicolae, 2008)] are based on multinomial models (MMs) of haplotype frequencies instead of HMM. These methods are simpler and faster, but their imputation accuracy is generally lower than the accuracy of HMM-based methods (Marchini and Howie, 2008, 2010). Recently, researchers proposed a new MM-based imputation method, called BLIMP, which imputes genotypes/allele frequencies for unmeasured SNPs by using the conditional expectation formula for multivariate normal variates (Wen and Stephens, 2010). Regardless of their model usage, all these imputation tools require a two-stage procedure that (i) imputes subject-level genotypes at the unmeasured SNPs on the basis of genotypes at measured SNPs and a relevant reference population [e.g. 1000 Genomes (1KG) (Altshuler *et al.*, 2010)] and (ii) tests for association between imputed genotypes and phenotype of interest. However, this procedure requires access to subject-level genotypes, which are often unavailable.

To directly impute summary statistics while (i) substantially reducing the computational burden and (ii) retaining imputation accuracy, we propose Direct Imputation of summary STatistics (DIST). DIST avoids the imputation of subject-level genotypes by directly applying, to unmeasured SNP statistics, the classical conditional expectation formula for multivariate normal variates.

## 2 SOFTWARE

DIST imputes the statistics at the unmeasured SNPs in a prediction window as a function of (i) the statistics at measured SNPs in a larger window, henceforth denoted as extended window, and (ii) the correlation matrix of both measured and unmeasured statistics, as estimated from a relevant reference dataset. Similar to BLIMP (Wen and Stephens, 2010), DIST uses the conditional mean formula for multivariate normal variates. Unlike BLIMP, DIST applies the formula (i) directly to summary statistics and (ii) under the null hypothesis, i.e. the hypothesis under which the distribution of all statistical tests is computed. This novel application allows the imputation of summary statistics without imputing subject-level genotypes (see Section 1.1 in Supplementary Material). To reduce computation time, DIST is implemented in C++, which ensures that it can be easily used under Linux, Windows, MacOS etc. DIST takes as input a file containing the (normally distributed) GWAS/meta-analysis summary statistics (see Section 1.5 of Supplementary Material for more information on obtaining the normally distributed statistics). It provides a command line interface with various options for specifying the number of measured SNPs to be contained in (i) the prediction window and (ii) each side region of the extended window, and so forth (Supplementary Table S1).

## 3 RESULTS

**Fig. 1.**
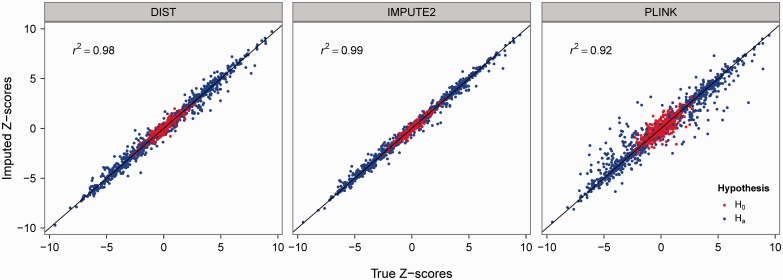
Imputed *Z*-scores as a function of the true *Z*-scores by imputation method (strip), under the null (red) and the alternative (blue) hypothesis

We compare the performance of DIST with the performance of typical HMM and MM methods. Given the speed and accuracy of SHAPEIT phasing (Delaneau *et al.*, 2012), we chose IMPUTE2 as the representative for the HMM-based methods. Given its wide availability, we chose PLINK as the representative for the MM-based methods. Thus, we compare the performance of DIST, IMPUTE2 and PLINK (at default settings) to predict 99 imputed height SNPs in 25 realistic simulations under both the null and the alternative hypothesis (Fig. 1). The phenotypes (height) of 5000 subjects are simulated as a function of the effects at the 180 significant SNPs from the height meta-analysis (Lango *et al.*, 2010) (Section 2 in Supplementary Material). Imputations used Europeans in 1KG as the reference sample and were performed on a single Linux machine (Intel Xeon 2.67 GHz processor and 64 GB of RAM).

The average accuracy of imputed *Z*-scores in the above 50 simulations, as measured by the squared correlation coefficient (*r*^2^) between imputed and true *Z*-scores, is high for DIST (0.98) and IMPUTE2 (0.99) and moderately high for PLINK (0.92) (Fig. 1). The average running time per simulation was 76 min for DIST, 270/965 min for imputing/pre-phasing for IMPUTE2 and 3971 min for PLINK. The maximum memory requirement was 52 MB for DIST, ∼9500 MB for IMPUTE2 and 5300 MB for PLINK. [DIST and IMPUTE2 were also used to impute the statistics for 5% SNPs missing at random on chromosome 22 of a dataset of 5000 subjects (which included the data described in the next paragraph); DIST required 33 min of running time and at most 283 MB of memory, and IMPUTE2 required 846/3437 min for imputation/pre-phasing and 9470 MB of memory.]

To impute untyped statistics, DIST requires only the joint correlation matrix for the statistics at typed and untyped SNPs. Because this matrix does not depend on the relationship between subjects in the study, unlike genotype imputation methods, DIST can be readily used to impute statistics for family association studies. We illustrate this advantage by applying the method to a proprietary Irish alcohol dependence study sample consisting of 1755 controls and 710 cases from 431 Irish families. The subjects were genotyped using Affymetrix 6.0 SNP array, and the association statistics were computed using MQLS (Thornton and McPeek, 2007). To impute unmeasured SNPs, we used UK10K (www.uk10k.org) as the reference panel (Supplementary Fig. S2).

## 4 CONCLUSIONS

DIST is a novel tool for direct imputation of summary statistics at untyped SNPs. When compared with genotype imputation methods, DIST (i) does not need access to subject-level genotypes, (ii) provides comparable imputation accuracy while substantially shortening the running time and (iii) can be readily applied to family association statistics. Consequently, DIST is useful for investigators who need fast and fairly accurate access to imputation-based *P*-values but (i) do not have access to subject-level genotypes, (ii) do not want to go through the laborious process of imputing subject-level genotypes or (iii) have association statistics coming from (large) pedigree data. Unlike genotype imputation methods, as the available reference panels are increasing in size, DIST can avoid incurring large increases in running time/memory by storing the local correlation structures into pre-computed tables.

When compared with genotype imputation methods, DIST uses a smaller imputation window and requires that study and reference populations to be well matched. Thus, when access to subject-level genotypic data is available, genotype imputation methods are likely to outperform DIST (i) for regions with long-range linkage disequilibrium, e.g. major histocompatibility complex locus, and (ii) when the study and the reference populations are not well matched. Consequently, whenever possible/appropriate, we recommend to follow-up DIST signals using a genotype imputation method.

*Funding*: Virginia Commonwealth University start-up fund (to S.A.B.)

*Conflict of Interest*: none declared.

## Supplementary Material

Supplementary Data
