# Supplementary material for: DIST: direct imputation of summary statistics for unmeasured SNPs
Source: Bioinformatics. 2013 Aug 28;29(22):2925–7. doi: 10.1093/bioinformatics/btt500 (PMC3810851; doi:10.1093/bioinformatics/btt500)
Supplement: Supplementary Data [file supp_29_22_2925__index.html]

DIST: Direct imputation of summary statistics for unmeasured SNPs — DIST: direct imputation of summary statistics for unmeasured SNPs — DIST: direct imputation of summary statistics for unmeasured SNPs — Supplementary Data 

# DIST: direct imputation of summary statistics for unmeasured SNPs

## Supplementary Data

files

**Files in this Data Supplement:**

- Supplementary Data - doc file
