# Supplementary material for: DIST: direct imputation of summary statistics for unmeasured SNPs
Source: Bioinformatics. 2013 Aug 28;29(22):2925–7. doi: 10.1093/bioinformatics/btt500 (PMC3810851; doi:10.1093/bioinformatics/btt500)
Supplement: Supplementary Data [file supp_btt500_supplementary_material.doc]

Table S1. Command line options currently implemented in DIST

| Option | Short Flag | Parameter | Default | Description |
| --- | --- | --- | --- | --- |
| --version | -v | none | none | Prints version information |
| --help | -h | none | none | Outputs a full description of all DIST options. |
| --reference | -r | filename | none | The filename of the reference data set. |
| --output | -o | filename | out.dst | The output filename. This file has a header and is space-delimited. Rows correspond to SNPs and columns to variables. |
| --chromosome | -c | positive integer between 1 and 22 | none | The chromosome number. |
| --num-pre | -n | positive integer | 1 | The number of measured SNPs to be contained in the prediction window (For more details, see sections 1.1 and 1.4). |
| --num-ext | -m | positive  integer | 50 | The number of measured SNPs included in the extended window to the left and right of the prediction window (For more details, see sections 1.1 and 1.4 below). |
| --info-cutoff | -i | subunitary  decimal | 0.99 | The information cutoff value for numerical integration. If information of an imputed SNP falls below it, DIST computes the imputed p-value by using the numerical integration method (See section 1.2 below). |
| --zscore-cutoff | -z | decimal | 3.0 | The absolute value for z-score cutoff for numerical integration. If the absolute value of the imputed z-score rises above this value and the corresponding imputation information falls below a user-defined cutoff value for imputation information (0.99 by default), DIST computes the imputed p-value by using the numerical integration method (See section 1.2 below). |
| --num-quant | -q | positive integer | 1,000 | The number of equally distant quantiles used for the numerical integration of the expected p-value for imputed statistics (See section 1.2 below). |

**1. Methods**

**1.1 Statistical method using conditional expectation formula**

DIST imputes normally distributed statistics of missing SNPs in a GWAS/meta-analysis in a direct manner, i.e. without imputing individual level genotypes, based on the classical formula for conditional expectation of multivariate normal variates. More specifically, let $Z_{1}=[\mathrm{z}_{i}]_{r\times1}$ be the vector of normally distributed statistics for all $r$ unmeasured SNPs in the prediction window containing $n$ measured SNPs. Let $Z_{2}=[\mathrm{z}_{i}]_{s\times1}$ be the vector of statistics for all $s$($=n+2m$) measured SNPs in the extended window, which includes i) the prediction window and ii) two regions, each containing $m$ measured SNPs, on both sides of the prediction window. Under the null hypothesis ($\text{H}_{0}$), of no association between genotypes and phenotype, $Z=\left[\begin{matrix} Z_{1} \\ Z_{2} \end{matrix}\right]$ is asymptotically distributed as a multivariate normal distribution, i.e. $Z\sim N(\left[\begin{matrix} 0 \\ 0 \end{matrix}\right],\text{ }\Sigma=\left[\begin{matrix} \Sigma_{11} & \Sigma_{12} \\ \Sigma_{21} & \Sigma_{22} \end{matrix}\right])$, where $\Sigma$ is the variance-covariance/correlation matrix having unitary diagonal entries. Thus, by using the conditional expectation formulas, $Z_{1}$ can be estimated by

$$\hat{\mu}=E(Z_{1}| Z_{2})=\Sigma_{12}(\Sigma_{22})^{-1}Z_{2}$$

and its variance-covariance matrix of $\hat{\mu}$, is estimated as

$\hat{\sigma}^{2}=Var(Z_{1}| Z_{2})=\Sigma_{11}-\Sigma_{12}(\Sigma_{22})^{-1}\Sigma_{21}$.

The diagonal of $\hat{\sigma}^{2}$ is the variance, i.e.uncertainty, of the $\hat{\mu}$estimator. It conveys information about the prediction accuracy. Given that the imputation field works with the complement of this variance, the diagonal elements of $I-\hat{\sigma}^{2}$ can be viewed as imputation information measures of $\hat{\mu}$. Consequently, each imputed value in $\hat{\mu}$ will be become more accurate as the information gets closer to 1.

Because under $\Sigma$ equals the correlation of genotypes, the variance-covariance matrix of the statistics can be estimated by the sample correlation matrix of the relevant genotypes in a reference data set, e.g. the 1000 Genomes Project (1KG) data set (Altshuler et al., 2010). To avoid inaccurate predictions due to very noisy correlation estimates and, thus, control the type I error in subsequent analyses, we found desirable to impute only SNPs having at least 10 (25) minor alleles in 1KG (UK10K- www.UK10K.org). While this recommendation might seem restrictive, these noisy estimates also affect haplotype frequency estimation required by the genotype imputation methods, as evidenced by the rare SNPs being well known as hard to impute (Li et al., 2011). Even more, the amount of omitted SNPs will rapidly decrease with the expected increase in the size of reference panels.

**1.2 Computing the p-value associated with the imputed statistics**

If $Z_{1}$ corresponds to measured SNPs, the p-value vector associated with $Z_{1}$ would be computed as $p_{1}=2(1-\Phi(|Z_{1}|))$. Given that $Z_{1}$ is imputed by $\hat{\mu}$, a naive way to compute p-values is

|  | $\hat{p}_{1}=2(1-\Phi(\vert\hat{\mu}\vert))$ | (1) |
| --- | --- | --- |

and DIST provides these p-values.

However, the naive calculation does not take into account the uncertainty/variability ($\hat{\sigma}^{2}$) of the conditional mean estimator, $\hat{\mu}$. Thus, to take into account this uncertainty and, thus, improve the accuracy of DIST p-value prediction, it is more desirable to compute the p-value of the imputation estimator as the expectation of the naive p-value over the distribution of the $\hat{\mu}$ estimate, i.e.

|  | $\text{E(}p_{1}\vert Z_{2})=\text{E}_{\hat{\mu},\hat{\sigma}^{2}}\left(2(1-\Phi(\vert Z_{1}\vert))\right)$. | (2) |
| --- | --- | --- |

Unfortunately, equation (2) does not have a closed form solution. Consequently, we solve (2) via a numeric integration method which uses $k$ equally distant quantiles ($k=1000$ by default). In more details, let $q_{\text{i}}\text{, i=1, … , }k$ be the equally distant quantiles from the $\text{Ν(}\hat{\mu}\text{, }\hat{\sigma}^{2}\text{)}$ distribution, i.e. $\text{F}_{\hat{\mu},\hat{\sigma}^{2}}(q_{\text{i}})=\frac{\text{i}}{k+1}$ (for each coordinate), where $\text{F}$ is the cumulative distribution of the conditional mean estimates. With this notation,

|  | $\text{E(}p_{1}\vert Z_{2})\approx\frac{\sum_{\text{i=1}}^{k} 2(1-\Phi(\vert q_{i}\vert))}{k}$. | (3) |
| --- | --- | --- |

When the information is high, i.e. $\hat{\sigma}^{2}$ is close to zero**,** $\text{E(}p_{1}|Z_{2})$ is close to the naive p-value estimator. However, when the information is low (and the uncertainty of the imputation is high), $\text{E(}p_{1}|Z_{2})$ can be far below the naive p-value (in green) estimator (see Fig. S1). (Thus, while the non-naive p-value adjustment from (3) controls the type I error, it is likely achieving this feat at the expense of an increased type II error.) If the information at an unmeasured SNP is lower than a user-specified cutoff information, 0.99 by default, and the absolute value of the Z-statistics exceeds a threshold, 3 by default, DIST imputes the p-value of the unmeasured SNP using equation (3). Otherwise, DIST provides only the naive estimator from (1).


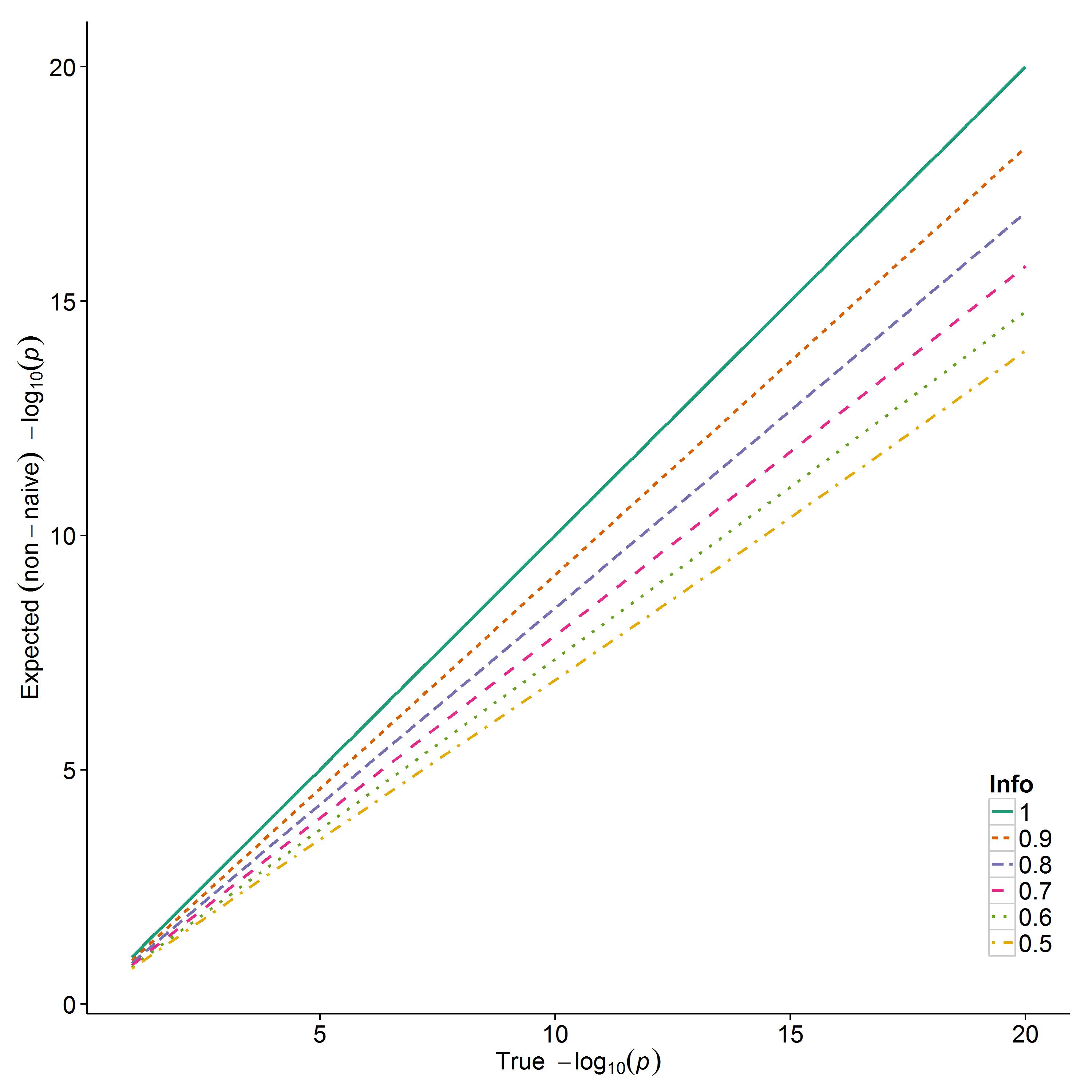


Figure S1. The expected non-naive $-\log_{10}(p)$ as a function of the true $-\log_{10}(p)$ and information.

**1.3 Rank deficiency**

Currently, reference populations have a small number subjects. Consequently, the sample correlation matrix may suffer from rank deficiency and biased estimates. Unlike Li and Stephens model-based approach (Li and Stephens, 2003; Wen and Stephens, 2010), we alleviate rank deficiency i) by restricting the number of measured SNPs, $s$, in the extended window to be less than the sample size in the panel and ii) regularizing $\Sigma_{11}$ and $\Sigma_{22}$, i.e. the sample correlation matrices of unmeasured and measured summary statistics, to be positive definite (by requiring all eigenvalues to be above a small positive threshold, e.g. 10^-5^). For instance, for 1000 Genomes (1KG) European reference panel with 381 subjects, we currently restrict the number of measured SNPs, $s$, in the extended window to be less than 381. For UK10K dataset having 2,432 subjects, $s$ is restricted to be less than 2,432. To alleviate the rank deficiency problem, whenever possible, we plan to increase the reference panel we use, e.g. by combining 1KG European sample with UK10K.

**1.4 The optimal number of measured SNPs in the extended window**

Unlike other genotype imputation methods using a sliding window of constant size (in Mbp), the length of the extended window (sliding window) of DIST is determined by the number of measured SNPs. Consequently, the length of this window varies when measured in Mbp. However, for our chosen way to measure the window length, is there an optimal window size? Intuitively, a small extended window might not use all the information in the correlation data and, given that the conditional expectation formula is in fact a regression model, a very large window might overfit the same data. To determine the optimal length, $s$, for the extended window, we performed a series of experiments involving the simulated data sets described in the main manuscript. We empirically assessed the correlation coefficient between 1KG imputed Z-scores and their true Z-scores by varying the number of measured SNPs in the prediction window, $n$, and the number of measured SNPs in each flanking area of the window, $m$. For our current implementation, the highest accuracy occurred when $n=1$ and $m=50$, i.e. $s=101$ (data not shown). Consequently, DIST uses these numbers as default.

**1.5 Converting statistics reported in GWAS-type studies into normally distributed statistics (Z-scores)**

Many GWAS studies report $\text{χ}^{2}$ statistics with one degree of freedom (df) along with odds ratios/slope coefficients for the reference (tested) alleles. (If only p-values are available, these can be transformed into $\text{χ}^{2}$ statistics using the inverse cumulative distribution function of the $\text{χ}^{2}$ statistics with one df.) To be used as input to DIST, these $\text{χ}^{2}$ statistics need to be converted into normally distributed statistics (Z-scores). This can be accomplished, for instance, by taking i) the absolute value of the Z-score to be the square root of the $\text{χ}^{2}$ statistic and ii) the sign of the Z-score to be a) the sign of reported log odds ratio for binary traits or b) the sign of the reported slope coefficients for quantitative traits. While the method can be easily extended to work with the unsigned $\text{χ}^{2}$ statistics, under the null hypothesis the correlation between these $\text{χ}^{2}$ statistics is (easily) computed to be the square of the correlation between genotypes. Consequently, due to the decreased correlation, when compared to methods using the information in the sign of Z-scores, the methods not using this information should lose imputation accuracy. This loss of accuracy should be especially pronounced at SNPs yielding information not very close to one.

**2. Realistic simulations based on height meta-analysis results**

These simulations are based on i) genotypes from around 2,500 unrelated control subjects from UK10K project and ii) effect sizes/coefficients, $c_{\text{j}}\text{, j}=1,\text{ }...\text{ },180$, of the significant signal SNPs from the height meta-analysis (Lango et al., 2010). For each simulation, the $n=5,000$ subjects are simulated by drawing at random, for each chromosome number, the genotypes of one subject from the UK10K sample. In more detail, for the $\text{i}^{th}$ subject, let $\text{Y}_{\text{i}}$, be the phenotype, $\text{X}_{\text{ij}},\text{ j}=1,\text{ }...\text{ },180$, be the genotype at the height significant loci. For a desired quantity, $h^{2}$, the phenotype is obtained as: $\text{Y}_{\text{i}}=\sum_{\text{j}=1}^{180} c_{\text{j}}\text{X}_{\text{ij}}+\varepsilon$, where the independent error term, $\varepsilon$, is normally distributed with mean $0$ and variance $1-h^{2}$. To obtain strong alternative signals at height SNPs, for the simulations with results presented in Fig. 1 from the main manuscript we used $h^{2}=0.4$. To realistically simulate an 1KG imputation process, we use the SNPs found on the Illumina 1M panel to impute the 99 significant height SNPs which were i) not found in this genotyping panel but ii) found on the 1KG imputation panel.


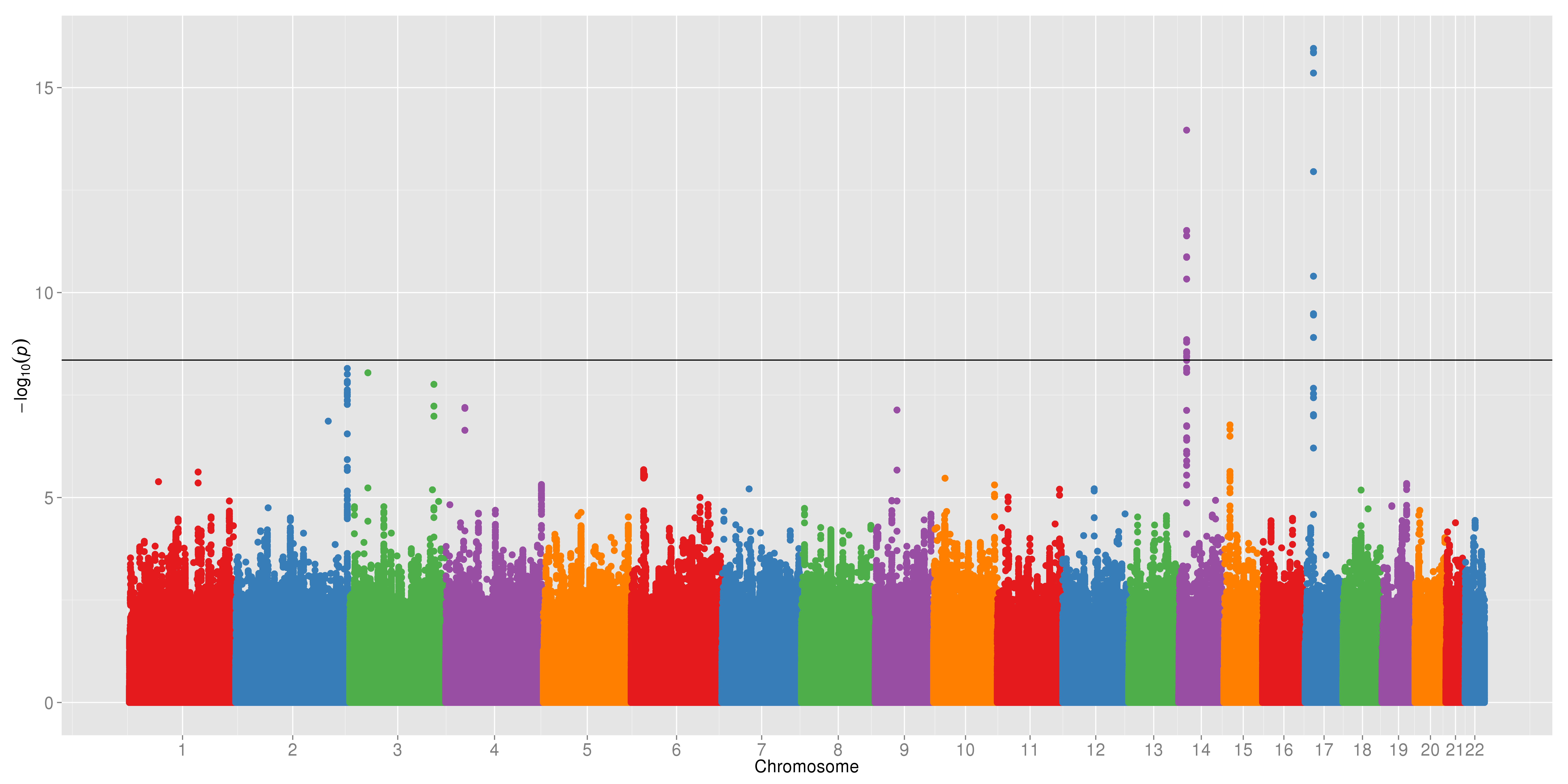


Figure S2. Manhattan plot of the $-\log_{10}(p)$ for testing the association between genotype and the alcohol dependence in the Irish Alcohol Dependence Study. Summary statistics at untyped SNPs were imputed by DIST using UK10K reference panel after filtering out SNPs with UK10K minor allele frequencies below 0.5%.

Reference List

Altshuler,D.L. et al. (2010) A map of human genome variation from population-scale sequencing. *Nature*, 467, 1061-1073.

Lango,A.H. et al. (2010) Hundreds of variants clustered in genomic loci and biological pathways affect human height. *Nature*, 467, 832-838.

Li,L. et al. (2011) Performance of Genotype Imputation for Rare Variants Identified in Exons and Flanking Regions of Genes. *Plos One*, 6.

Li,N. and Stephens,M. (2003) Modeling linkage disequilibrium and identifying recombination hotspots using single-nucleotide polymorphism data. *Genetics*, 165, 2213-2233.

Wen,X.Q. and Stephens,M. (2010) Using Linear Predictors to Impute Allele Frequencies from Summary Or Pooled Genotype Data. *Annals of Applied Statistics*, 4, 1158-1182.
